# Urine Fetuin-A is a biomarker of autosomal dominant polycystic kidney disease progression

**DOI:** 10.1186/s12967-015-0463-7

**Published:** 2015-03-30

**Authors:** Nathalie Piazzon, Florian Bernet, Linda Guihard, Wouter N Leonhard, Séverine Urfer, Dmitri Firsov, Hassib Chehade, Bruno Vogt, Sophia Piergiovanni, Dorien JM Peters, Olivier Bonny, Daniel B Constam

**Affiliations:** Ecole Polytechnique Fédérale de Lausanne (EPFL), Bâtiment SV ISREC, Station 19, Lausanne, Switzerland; Department of Pharmacology and Toxicology, University of Lausanne (UNIL), Quartier UNIL-CHUV, Lausanne, Switzerland; Service of Nephrology, Lausanne University Hospital (CHUV), Lausanne, Switzerland; Department of Human Genetics, Leiden Univ. Medical Center, Leiden, The Netherlands; Department of Pediatrics, Division of Pediatric Nephrology, Lausanne University Hospital (CHUV), Lausanne, Switzerland; Department of Nephrology and Hypertension, Inselspital, Bern, Switzerland

**Keywords:** Fetuin-A, Urine, ADPKD, Biomarker, ELISA

## Abstract

**Background:**

Autosomal dominant polycystic kidney disease (ADPKD) is a genetic disorder characterized by numerous fluid-filled cysts that frequently result in end-stage renal disease. While promising treatment options are in advanced clinical development, early diagnosis and follow-up remain a major challenge. We therefore evaluated the diagnostic value of Fetuin-A as a new biomarker of ADPKD in human urine.

**Results:**

We found that renal Fetuin-A levels are upregulated in both *Pkd1* and *Bicc1* mouse models of ADPKD. Measurement by ELISA revealed that urinary Fetuin-A levels were significantly higher in 66 ADPKD patients (17.5 ± 12.5 μg/mmol creatinine) compared to 17 healthy volunteers (8.5 ± 3.8 μg/mmol creatinine) or 50 control patients with renal diseases of other causes (6.2 ± 2.9 μg/mmol creatinine). Receiver operating characteristics (ROC) analysis of urinary Fetuin-A levels for ADPKD rendered an optimum cut-off value of 12.2 μg/mmol creatinine, corresponding to 94% of sensitivity and 60% of specificity (area under the curve 0.74 ; p = 0.0019). Furthermore, urinary Fetuin-A levels in ADPKD patients correlated with the degree of renal insufficiency and showed a significant increase in patients with preserved renal function followed for two years.

**Conclusions:**

Our findings establish urinary Fetuin-A as a sensitive biomarker of the progression of ADPKD. Further studies are required to examine the pathogenic mechanisms of elevated renal and urinary Fetuin-A in ADPKD.

**Electronic supplementary material:**

The online version of this article (doi:10.1186/s12967-015-0463-7) contains supplementary material, which is available to authorized users.

## Background

Autosomal dominant polycystic kidney disease (ADPKD) is the most common inherited kidney disease, affecting as many as 1 in 1000 individuals world-wide, but with variable course and prognosis [1-3]. It is caused by mutations in the *polycystic kidney disease (PKD)-1* or less frequently in the *PKD2* gene [4-7]. ADPKD is characterized by the progressive development of numerous large fluid-filled cysts especially in the kidneys over a period of decades [8]. Cystic growth results in dramatic kidney enlargement, and it induces reactive interstitial inflammation and fibrosis, leading frequently to end stage renal disease (ESRD) [9]. However, important unresolved issues remain in the diagnosis and follow-up of ADPKD. In particular, what determines the rate of cyst progression in patients is unclear, and diagnostic tools to predict disease outcome are elusive. Diagnosis is usually established by renal imaging (ultrasonography, CT-scan or MRI) when there is a positive family history [7]. However, cysts may only appear late in the course of the disease, creating a diagnostic gap. Direct genetic analysis is feasible, but remains challenging owing to the large size, complex genomic structure and allelic heterogeneity of *PKD1* and *PKD2* genes [10,11]. Disease progression is usually assessed by repeated analysis of plasma creatinine levels as readout of glomerular filtration rates (GFR). However, plasma creatinine levels only start rising when the disease is already well advanced. To improve diagnosis and early follow-up of ADPKD, current efforts focus on renal volume assessment by MRI or CT-scan and on non-invasive urine biomarkers. Assessment of renal volume allows an earlier follow-up of the disease and the management of associated symptoms such as hypertension [12]. Only few candidate biomarkers have been identified, including albuminuria, β2-microglobulin [13,14], neutrophil gelatinase-associated lipocalin (NGAL) [15] and monocyte chemotactic protein 1 (MCP-1) [16,17]. With the notable exception of albuminuria [18,19], these remain to be validated.

Future diagnostic and innovative therapeutic approaches may be guided by insights from rodent models of ADPKD with spontaneous or engineered mutations [20]. Among such models, mouse kidneys lacking *Pkd1* are arguably the most disease-relevant since human *PKD1* is mutated in 85% of ADPKD patients [21]. Interestingly, conditional knockout of a targeted *Pkd1* allele (*Pkd1*cKO) in mouse kidneys before or after postnatal day 12–14 revealed that the susceptibility to cystic growth dramatically decreases after day 13, coincident with a metabolic switch and a sudden decline of cell proliferation at this stage of postnatal development [22-25]. Transcriptome profiling of *Pkd1*cKO kidneys revealed that even the aggressive early-onset disease is initiated independently of changes in gene expression levels above 2-fold, and with less than 100 genes de-regulated [24]. Therefore, it may be difficult to identify sensitive markers linked to the etiology of ADPKD that are significantly deregulated at the transcriptional level.

Renal cysts also develop in mice and humans carrying mutations in the *Bicc1* gene that encodes the cytoplasmic RNA-binding protein Bicaudal-C [26-29]. *Bicc1* expression partly depends on *Pkd1* [30] and in turn stimulates the translation of *Pkd2* mRNA [27], indicating that Bicaudal-C mediates critical polycystin functions. A candidate search for direct targets revealed that Bicc1 binds adenylate cyclase-6 (AC6) mRNA and reduces its translation [27]. AC6 is likely a ADPKD-relevant target as it contributes to cyst formation in Pkd1-deficient mouse kidneys [31]. However, since AC6 or other known direct Bicc1 targets cannot be monitored non-invasively, we decided instead to initially screen for candidate biomarkers using gene expression profiling.

Here, we report that polycystic mouse kidneys induced by targeted deletion of *Pkd1* or *Bicc1* as well as the urine of ADPKD patients contain elevated levels of Fetuin-A. Fetuin-A (also known as α2-Heremans Schmid glycoprotein, AHSG, FETUA) is a multifunctional negative acute phase protein in blood plasma that regulates insulin signaling, bone resorption, and the precipitation of calciprotein particles [32]. During development, FETUA is expressed in several tissues and organs, including the brain, liver, bone, kidney, and respiratory and cardiovascular systems [33], whereas in adults, its expression normally is restricted to the liver [34]. Despite the absence of *FETUA* mRNA, the protein has been detected in proximal tubule epithelial cells of adult rat kidneys by immunostaining, and this staining can be inhibited by blocking megalin-mediated endocytosis [35]. Thus, Fetuin-A may enter proximal tubule cells by reabsorption from the tubule lumen after passing from plasma through the glomerular slit diaphragm [35,36]. We show that Bicc1 mutant cystic kidneys fail to retain the protein and instead release it into urine. We show that urinary Fetuin-A levels are also elevated in patients with ADPKD compared to healthy volunteers. Our findings reveal a new trait shared among ADPKD patients and the *Bicc1* mouse model, and they suggest that Fetuin-A is a promising new disease biomarker that should be considered for further prospective studies and to investigate its potential role in ADPKD pathogenesis.

## Results

### Upregulation of Fetuin-A in two mouse models of polycystic kidney disease

**Figure 1:**
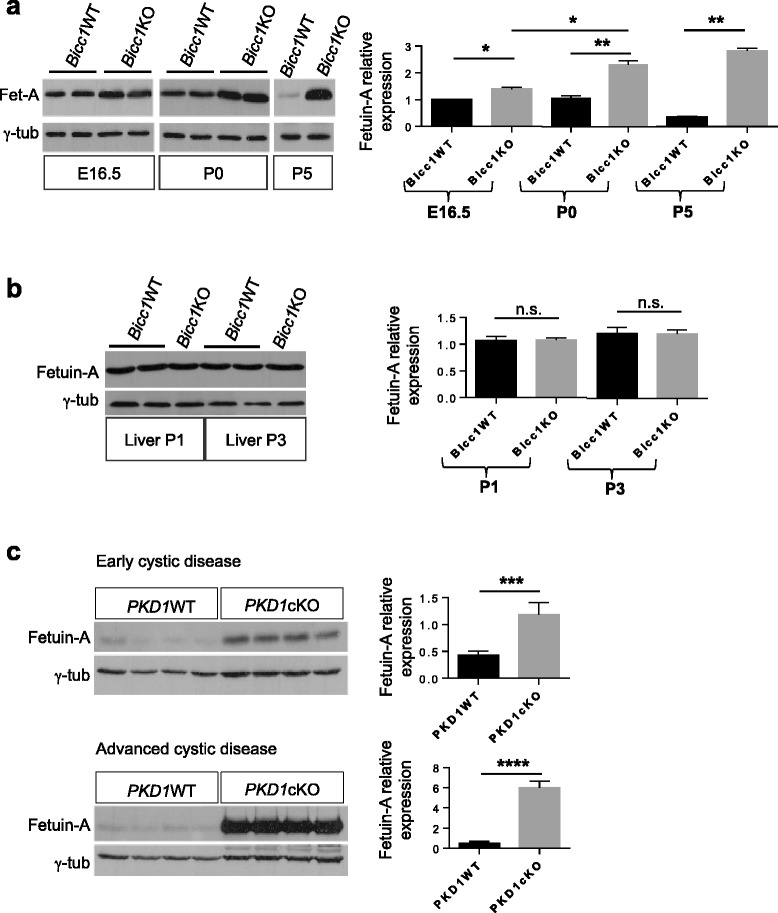
**Expression of Fetuin-A in mouse models of polycystic kidney disease. (a, b)** Western blot analysis of Fetuin-A in **(a)** kidney and **(b)** liver extracts of the indicated genotypes. **(c)** Western blots of Fetuin-A in early stages of cyst formation (3 weeks, n = 4 for each genotype) or advanced cystic kidneys (9 weeks, n = 4 for each genotype) of wild-type or KspCad-Cre^ERT2^; *Pkd1* ^fl/fl^ kidneys after tamoxifen administration at postnatal days 10–12. Signal quantification by Image J is shown on the right after normalization to loading control (γ-tubulin). Error bars represent standard deviation. n.s.: non-significant.

Insights into disease pathogenesis and identification of biomarkers may be accelerated by genome-wide transcriptome analysis [37]. To this end, total RNA from 3 *Bicc1*KO embryos and 3 wild-type (WT) control littermates was isolated shortly after the onset of cyst formation and subjected to Affymetrix cDNA array hybridization. Array analysis only revealed very few and modest changes at the level of gene transcription (less than 70 genes being changed more than 1.5-fold, data not shown). Among these changes was a 3.5-fold upregulation of the *FETUA* transcript (p = 0.006). Consistently, Western blot analysis of renal extracts at E16.5 (i.e. during onset of cyst formation), P0 and P5 showed increasing expression of Fetuin-A protein with the expected apparent molecular weight of 59 kDa in *Bicc1*KO compared to WT (Figure 1a). While Fetuin-A is mainly synthesized in the liver after birth [34], Western blot analysis of liver extracts at P1 and P3 revealed no difference in its expression in *Bicc1*KO compared to the WT (Figure 1b). This result suggests that Fetuin-A expression is increased in *Bicc1*KO kidneys, rather than systemically.

To verify whether the increase in renal Fetuin-A was related to cyst formation, we analyzed kidneys carrying a conditional *Pkd1* allele that was deleted by tamoxifen-inducible KspCad-CreERT2 [23,25]. Compared to WT, Fetuin-A was upregulated up to 2.7-fold (p < 0.001) during an early stage of cyst formation, and 7.8-fold (p < 0.001) in animals with advanced cysts (Figure 1c). These results suggest a possible correlation between the level of Fetuin-A and the progression of the disease.

### Fetuin-A levels are low in cyst-lining cells, but increase in urine of Bicc1KO mice

**Figure 2:**
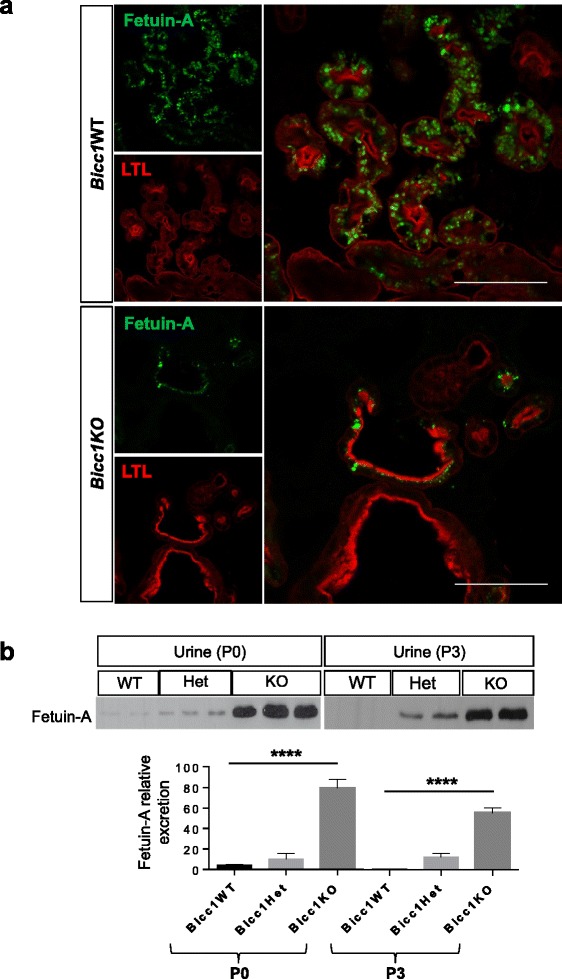
**Localization and excretion of urinary Fetuin-A in newborn kidneys. (a)** Frozen sections of WT (n = 2) and *Bicc1*KO newborn kidneys (n = 4) labeled with anti-Fetuin-A antibody (green), and *Lotus tetragonolobus* lectin (LTL, red). *Bicc1* heterozygotes (n = 2) were indistinguishable from WT (not shown). Scale bars: 100 μm. **(b)** Western blot analysis of urinary Fetuin-A excretion of the indicated genotypes at stages P0 and P3. Signal quantification by ImageJ is shown below. Error bars represent standard deviation.

To obtain further insight into the regulation of Fetuin-A, we examined its distribution in WT and *Bicc1*KO newborn kidneys by immunostaining. Co-labeling with *Lotus tetragonolobus* lectin, which specifically labels proximal tubules [38] revealed Fetuin-A expression in cortical epithelial cells of newborn wild-type kidney (Figure 2a). Fetuin-A was neither detected in the medulla nor in the renal pelvis (Additional file 1: Figure S1), concurring with data obtained in rat kidneys [35]. Paradoxically, despite the marked elevation of Fetuin-A in total extracts of *Bicc1*KO newborn kidneys above WT control (Figure 1a), immunolabelling only detected very sparse Fetuin-A staining in few cyst-lining cells in proximal tubules (Figure 2a). To assess whether Fetuin-A accumulates in the urine, we collected urine samples from the bladder at P0 or P3. Compared to heterozygous and WT litter mates, urine from *Bicc1*KO showed an up to 60-fold increase in Fetuin-A levels (n = 5/5, p < 0.005) (Figure 2b). We conclude that cystic *Bicc1*KO kidneys enrich Fetuin-A in the urine.

### Levels of urinary Fetuin-A correlate with disease progression in ADPKD patients

**Figure 3:**
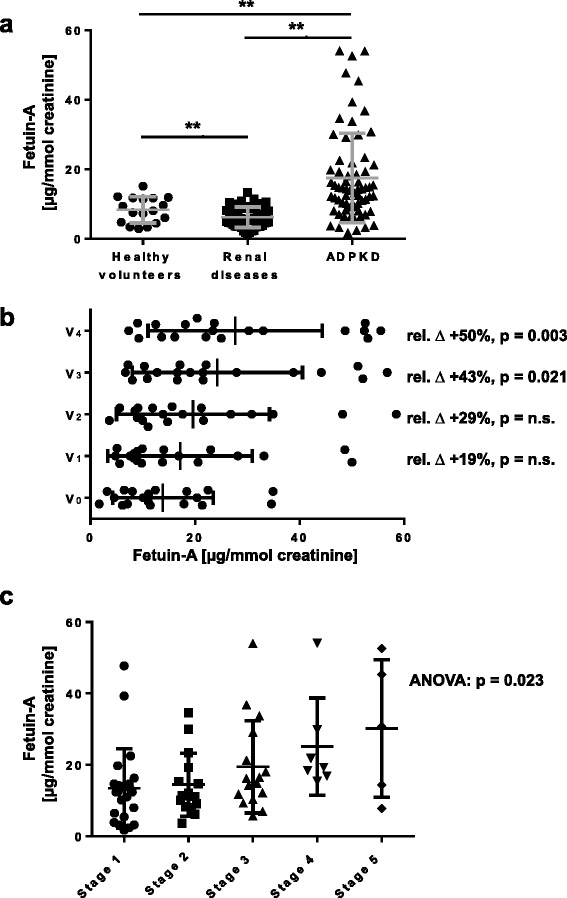
**Evaluation of Fetuin-A as a biomarker for ADPKD. (a)** ELISA quantification of Fetuin-A levels normalized to creatinine in urine of ADPKD (n = 66), healthy volunteers (n = 17) and control patients with various renal diseases (n = 50). Solid lines indicate mean. **(b)** Evolution of urinary levels of Fetuin-A normalized to creatinine during 5 visits (V) every 6 months in 19 early ADPKD patients. Average relative change (∆, %) and p-values are indicated. **(c)** Urinary levels of Fetuin-A normalized to creatinine stratified by CKD stages evaluated based on eGFR values. Stage 1, eGFR = 115 ± 10 ml/min/1.73 m^2^ (range, 96–129), n = 23; stage 2, eGFR = 77 ± 8 ml/min/1.73 m^2^ (range, 61–89), n = 15; stage 3, eGFR = 45 ± 9 ml/min/1.73 m^2^ (range, 31–59), n = 16; stage 4, eGFR = 21 ± 2 ml/min/1.73 m^2^ (range, 18–25), n = 7 and stage 5, eGFR = 11 ± 1 ml/min/1.73 m^2^ (range, 10–14), n = 5. Solid lines indicate mean. Significance was calculated by one-way ANOVA (p = 0.023).

**Figure 4:**
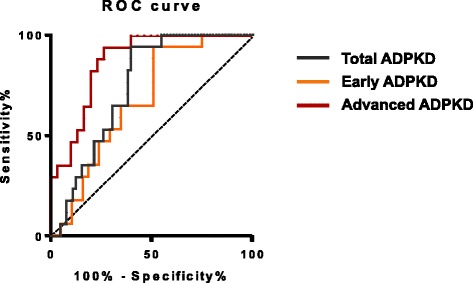
**Receiver Operating Characteristic (ROC) curves of urinary Fetuin-A as a biomarker for detection of ADPKD.** Receiver Operation Characteristic (ROC) curves. The area under the ROC curve was 0.74 (95% CI, 0.6397 to 0.8505, p < 0.05) for ADPKD diagnosis, 0.67 (95% CI, 0.5276 to 0.8143, p < 0.05) for early ADPKD diagnosis, 0.87 (95% CI, 0.7720 to 0.9692, p < 0.05) for advanced ADPKD diagnosis.

We therefore evaluated urinary Fetuin-A as a potential ADPKD biomarker in humans. ELISA analysis was conducted on urine samples from 66 ADPKD patients, 17 healthy volunteers and 50 patients with renal diseases of other causes than ADPKD. Clinical characteristics and laboratory variables of patients are reported in Table 1 and Additional file 2: Table S1. Urinary levels of Fetuin-A normalized to creatinine were significantly higher in ADPKD patients (17.5 ± 12. 9 μg/mmol creatinine) compared to healthy volunteers (8.5 ± 3.8 μg/mmol creatinine) and to patients with various renal diseases other than ADPKD (6.2 ± 2.9 μg/mmol creatinine) (Figure 3a). To verify the expression of urinary Fetuin-A in individual urine samples by Western blotting, 11 samples from ADPKD patients and patients with various renal diseases other than ADPKD were randomly selected. The result demonstrated that urinary Fetuin-A levels were significantly augmented in ADPKD group, correlating with the ELISA analysis (Additional file 3: Figure S2). Moreover, consecutive analysis in 6 months intervals during 2 years in 19 patients with early-stage ADPKD (eGFR > 60 ml/min/1.73 m^2^) showed that urinary excretion of Fetuin-A increased progressively (p = 0.031, one-way ANOVA) (Figure 3b, Additional file 4: Figure S3a and Table 2). By contrast, eGFR did not significantly change over the same time period (Additional file 4: Figure S3b). This suggests that the levels of Fetuin-A in urine may be a more sensitive marker in determining disease progression than eGFR. Consistently, a significant correlation was found between chronic kidney disease (CKD) stages and the levels of urinary Fetuin-A in ADPKD patients (p = 0.024, one-way ANOVA) (Figure 3c, Additional file 4: Figure S3c and Table 3). To further validate this conclusion, we determined cut-off values discriminating ADPKD patients from normal volunteers using Receiver Operating Characteristics (ROC) curves (Figure 4). All areas under the curves (AUC) were significantly different from chance. Urinary Fetuin-A levels of advanced ADPKD (eGFR < 60 ml/min/1.73 m^2^) showed the highest AUC (0.87, p < 0.0001) and that Fetuin-A values at 12.2 μg/mmol creatinine distinguished patients from healthy controls with 94% of sensitivity and 74% of specificity. Fetuin-A values at 12.2 μg/mmol creatinine distinguished patients with early ADPKD (eGFR > 60 ml/min/1.73 m^2^) from healthy controls, but the sensitivity and specificity were 94% and 58%, respectively. These analyses yielded optimum cut-off values of 12.2 μg/mmol creatinine for the establishment of ADPKD diagnosis with a high sensitivity and a reasonable specificity.

**Table 1:** **Clinical characteristics and laboratory variables of patients subjected to ELISA analysis**

	**Healthy volunteers**	**Renal diseases other than ADPKD**	**ADPKD**		
			**Total**	**Early**	**Advanced**
Cases (n)	17	50	66	36	30
Age (Years)*	34.9 ± 11.1	65.3 ± 12.6	43.1 ± 17.2	31.9 ± 12.7	56.3 ± 11.5
Gender (n)**					
• Male	53 (9)	66 (33)	48 (32)	50 (18)	47 (14)
• Female	47 (8)	34 (17)	52 (34)	50 (18)	53 (16)
Pathologies (n)**					
• Diabetes	-	20 (10)	3 (2)	-	6 (2)
• Obesity (BMI > 30 kg/m^2^)	-	16 (8)	3 (2)	3 (1)	3 (1)
• Hypercholesterolemia	-	24 (12)	7 (5)	-	16 (5)
eGFR [mL/min/1.73 m^2^]*	N.A.	30.0 ± 15.1	71.8 ± 38.8	102.5 ± 20.6	35.4 ± 17.4
Proteinuria [g/ mmol creatinine]*	N.A.	0.621 ± 0.06	0.139 ± 0.05	0.101 ± 0.04	0.215 ± 0.02
High BP, (n)**	-	44 (22)	85 (56)	75 (27)	96 (29)
Fetuin-A (U) [μg/L]*	78.7 ± 39.5	39.2 ± 13.1	104.9 ± 58.7	89.1 ± 48.4	124.1 ± 64.7
Fetuin-A (U) [μg/mmol creatinine]*	8.5 ± 3.8	6.2 ± 2.9	17.5 ± 12.9	13.8 ± 9.6	21.8 ± 14.3

*Data are presented as mean ± SD. ADPKD, autosomal dominant polycystic kidney disease; eGFR, estimated glomerular filtration rate; N.A., not assessed; (U), urine.

**Values are in percentage. Absolute numbers are in brackets. High BP is defined as systolic BP >140 and/or diastolic BP >90 mmHg (mean of three measurements) or the need for anti-hypertensive medication.

**Table 2:** **Levels of urinary Fetuin-A correlate with ADPKD progression**

**Early ADPKD**	**V**_**0**_	**V**_**1**_	**V**_**2**_	**V**_**3**_	**V**_**4**_
Time (months)	0	6	12	18	24
Cases (n)	19	19	19	19	19
Fetuin-A [μg/L]*	71.6 ± 43.7	95.6 ± 47.8	114.3 ± 65.3	136.1 ± 56.7	150.7 ± 66.4
p-value	n.s.	n.s.	n.s.	0.014	0.0001
Fetuin-A [μg/mmol creatinine]*	13.9 ± 9.6	17.1 ± 13.8	19.6 ± 14.6	24.3 ± 16.3	27.7 ± 16.7
p-value	n.s.	n.s.	n.s.	0.021	0.003

*Data are presented as mean ± SD. One-way ANOVA followed by *Bonferroni’s* test was used for comparison (p < 0.05) for both Fetuin-A and Fetuin-A/Creatinine ratio. n.s., not significant.

**Table 3:** **Correlation between levels of urinary Fetuin-A and CKD stage of patients**

**CKD stage**	**1**	**2**	**3**	**4**	**5**
Cases (n)	23	15	16	7	5
Fetuin-A [μg/L]*	88.4 ± 42.4	90.6 ± 51.5	113.1 ± 63.7	152.1 ± 64.8	172.4 ± 22.8
p-value		n.s.	0.024	0.003	0.0001
Fetuin-A [μg/mmol creatinine]*	13.4 ± 11.1	14.5 ± 8.8	19.4 ± 12.9	25.1 ± 13.6	30.2 ± 19.3
p-value		n.s.	0.041	0.027	0.012

*Data are presented as mean ± SD. n.s., not significant.

The 66 ADPKD patients were stratified by CKD stages (1 to 5). The stages are based on estimated glomerular filtration rate (eGFR). One-way ANOVA followed by *Bonferroni’s* test was used for comparison (p < 0.05) for both Fetuin-A and Fetuin-A/creatinine ratio.

## Discussion

Improving early diagnosis and follow-up of ADPKD patients remains a major challenge, especially for disease management in the new era of future therapeutic interventions [39]. Ongoing efforts so far have mainly focused on imaging (CRISP consortium) [40] and on new disease biomarkers [41-44]. However, despite intense efforts, the development of non-invasive biomarkers is still in its infancy. Here, we found that two independent mouse models of ADPKD express elevated levels of Fetuin A in the kidney, and that average urinary Fetuin-A levels increase during disease progression in affected ADPKD patients.

Renal Fetuin-A levels increased in the urine of ADPKD patients compared to healthy volunteers, as well as in urine and total kidney extracts of the non-orthologous *Bicc1*^−/−^ mouse model. In view of the positive data from urine of patients, we did not attempt to extend our study to urine of conditionally *Pkd1*-deleted mutant mice. Nevertheless, analysis of whole kidneys confirmed that Fetuin-A is also upregulated in this orthologous mouse model, in keeping with a role as a disease-relevant marker. Importantly, the levels of urinary Fetuin-A in ADPKD patients correlated with the stage of renal insufficiency during disease progression. In good agreement, Fetuin-A has also been detected within ADPKD kidneys by mass spectrometry analysis of cyst fluid [45]. Together, these observations indicate that Fetuin-A is a sensitive biomarker of disease progression.

Fetuin-A initially attracted our attention because of an apparent upregulation detected by microarray gene expression profiling of embryonic *Bicc1*KO kidneys already at the early onset of cystic disease. However, local transcriptional de-regulation within the kidney could not be confirmed either by Taqman or SYBR Green qRT-PCR due to low baseline expression and substantial variability among samples both in wild-type and cystic newborn kidneys (unpublished observation). Furthermore, immunolabelling detected Fetuin-A in vesicles of wild-type proximal tubules as described previously in the rat [35], and this staining was nearly abolished in *Bicc1*KO, rather than being increased. Nevertheless, the levels of Fetuin-A detected by Western blot clearly increased in *Bicc1*KO compared to wild-type, both in total kidney extracts and in urine. Analysis of livers detected no systemic upregulation or increased hepatic production. Moreover, in a cohort of ADPKD patients that was followed during 24 months, we found that the levels of urinary Fetuin-A significantly increased without a corresponding increase in eGFR. Taken together, these findings suggest that Fetuin-A specifically accumulates in the urine of cystic kidneys, probably due to impaired renal reabsorption rather than increased filtration or secretion.

Fetuin-A is also secreted in the urine during advanced diabetic nephropathy [46]. However, the levels of urinary Fetuin-A in these patients correlate with microalbuminuria, indicating that Fetuin-A leaked through defective glomeruli and failed to be reabsorbed in proximal tubules. Fetuinuria in diabetic nephropathy thus likely has a different origin than in ADPKD. The levels of Fetuin-A in urine also increase during acute kidney injury (AKI), which can be induced by cisplatin or by ischemia-reperfusion [47]. During AKI, Fetuin-A was enriched both in the cytoplasm of damaged proximal tubule cells and in urine [47], similar to what we observed in cystic kidneys of *Bicc1* KO mice. By contrast, we detected no upregulation of Fetuin-A in the urine of patients with various renal diseases other than ADPKD. Also in urine of patients with kidney stones, a recent study reported a decrease in the levels of Fetuin-A, rather than an increase [48]. Thus, rather than being a general reaction to renal injury, elevated Fetuin-A in adult human kidneys appears to be part of a program that is linked to ADPKD.

Acknowledged limitations of this study include the absence of kidney volume assessment for quantifying the rate of disease progression, and that our patient group has not been stratified by genotype or extrarenal manifestations such as hypertension or liver cysts. Nevertheless, our finding that Fetuin-A is significantly upregulated in a majority of ADPKD patients and in mouse kidneys deficient in *Pkd1* or one of its downstream targets might become clinically useful to aid in diagnosis.

## Conclusions

In summary, our findings demonstrate that Fetuin-A production is upregulated in cystic kidneys of two different mouse models of ADPKD and excreted in the urine. In ADPKD patients, urinary Fetuin-A levels were significantly elevated, with a close correlation between fetuinuria and disease progression. While the precise mechanisms underlying the increase of Fetuin-A secretion in the kidney and its role in cystic disease remain to be determined, our findings establish urine Fetuin-A as a novel biomarker of the progression of ADPKD in humans. Further studies are warranted to examine the pathogenic mechanisms of elevated Fetuin-A and its role in the diagnosis of ADPKD.

## Methods

### Mice and mutant alleles

Mice heterozygous for a targeted null allele of *Bicc1* [29] were maintained on a C57BL/6 genetic background in individually ventilated cages at the EPFL animal facility. To induce a kidney-specific deletion of *Pkd1*, tam-KspCad-CreERT2; Pkd1^lox2–11/lox2–11^ mice orally received tamoxifen by gavage as described [23]. For early stage of cyst formation, mice were sacrificed 3 weeks after administration of 0.5 mg taxomifen at postnatal days 10–12. For late stage of cyst formation, mice were sacrificed 9 weeks after administration of 0.5 mg taxomifen at postnatal days 10–12. All animal experiments were approved by the Animal Care and Use Committee of Leiden University and by the Commission for Biotechnology in Animals of the Dutch Ministry of Agriculture, or by the Veterinary Service of the Swiss canton of Vaud.

### Clinical specimen collection

133 participants (66 ADPKD and 50 patients with various renal diseases and 17 normal volunteers) were enrolled in the ADPKD cohort of the division of Nephrology of the Lausanne University Hospital (CHUV) (Lausanne, Switzerland) prospectively between 2009 and 2014. Clinical data of all participants were collected. All patients were informed about the purpose of the study and gave their informed consent. This study was approved by the local research ethical board and conducted in accordance with the ethical standards of the Helsinki Declaration.

### Biochemical assays

Each human urine sample was directly collected into sterile plastic tube and then stored at −80°C for further analysis. Blood samples were immediately processed. Serum creatinine, osmolality, sodium, potassium, calcium, phosphate, BUN, uric acid and urine creatinine, osmolality, sodium, potassium, total proteins were measured at the central lab of the Lausanne University Hospital (Lausanne, Switzerland). Serum creatinine values were used to calculate estimated glomerular filtration rate (eGFR) using the CKD-Epidemiology Collaboration (CKD-EPI) equation [49].

### RNA isolation, library preparation and Affymetrix cDNA microarray hybridization

Kidney RNA was extracted using Trizol (Invitrogen) according to the manufacturer instructions. RNA concentration and purity were determined using a Nanodrop (Thermo Fisher, Waltham, MA). RNA integrity was assessed on a Bioanalyzer (Agilent, Santa Clara, CA). High-quality RNA samples (RNA Integrity Number ≥ 8) were used for library preparation.

### Indirect immunofluorescent labelling

Postnatal mouse kidneys were fixed in 4% paraformaldehyde in PBS for 1.5 hrs, rinsed with PBS, and soaked in 15% sucrose over night at 4°C and embedded in Optimum Cutting Temperature (OCT) compound on dry ice. Cryosections (8 μM) were permeabilised for 10 min with 0.2% Triton X-100 in PBS, blocked with PBS containing 1% BSA and with streptavidin-biotin blocking kit (Vector labs, #SP-2002). Biotinylated LTL (Vector labs, B-1325), 1:200, and goat anti-Fetuin-A antibody (1:300) were added over night at 4°C. Secondary antibody (anti-rabbit Alexa 488, Molecular Probes) and streptavidin pacific blue (Lifetechnologies) were incubated for 1 hr at 25°C at 1:800 and 1:400, respectively, in PBS containing 0.1% Triton X-100. Images were acquired with a 20× objective on a Leica LSM700 confocal microscope.

### Western blot analysis

Frozen kidneys were directly lysed in Laemmli buffer by ultrasonication on ice and centrifuged to remove debris while retaining cyst fluids [45] and urine. For Western blot analysis, urine samples were centrifuged at 3,500 × g for 5 min at 4°C. Proteins were separated by electrophoresis on a 7.5% sodium dodecyl sulfate-polyacrylamide gel and transferred onto nitrocellulose membranes (Amersham-Biosciences). The membranes were blocked for 1 h at 37°C in a solution of PBS containing 5% non-fat milk powder and 0.05% Tween-20 (PBS-T) and then incubated overnight at 4°C with goat polyclonal primary antibody against Fetuin-A (diluted 1:200; Santa Cruz Biotechnology Inc., USA) or mouse anti-γ-tubulin (diluted 1:1000; Sigma). Membranes were washed three times for 10 min in PBS-T and then incubated with the secondary antibodies peroxidase-conjugated anti-mouse or anti-rabbit (GE Healthcare) and anti-goat antibodies (Santa Cruz Biotechnology Inc., USA) at room temperature for 1 h. The proteins were detected using an enhanced chemiluminescence detection system (ECL-Direct systems RPN3000; Pierce Biotechnology, Inc., Rockford, IL, USA). Densitometric analysis of immunoblots and normalization to γ-tubulin expression was performed using Image J software.

### Enzyme-linked immunosorbent assay (ELISA)

Urine samples were centrifuged at 3,500 × g for 5 min at 4°C to remove debris prior to ELISA analysis. The concentrations of Fetuin-A in the urine samples were measured with a Fetuin-A (AHSG) Human ELISA kit (Abcam, Cambridge, UK). The assay was performed according to the instructions recommended by the manufacturer. The standard curve was created using lyophilized human Fetuin-A standard preparation supplied with the assay. Following the colorimetric reaction, the optical density was read at 450 nm using a spectrophotometer, and converted to concentrations in μg/L. Urine creatinine levels were measured at the central lab of the Lausanne University hospital using a Cobas-Mira analyzer (Roche). The levels of Fetuin-A were normalized according to urine creatinine concentrations (Fetuin-A: μg/mmol creatinine). Every sample was tested in duplicate.

### Statistical analysis

All data were collected and presented as mean ± standard deviation. Normal distribution of the data was assessed using Shapiro-Wilk test. Differences among two groups were compared by unpaired Student’s t-test. For comparison among multiple experimental conditions, a nonparametric one-way analysis of variance (ANOVA) followed by *Bonferroni’s test* for multiple comparisons was used. P < 0.05 was considered a statistically significant difference. Receiving operating curve (ROC) analyses were used to explore the diagnostic performance of urinary Fetuin-A (μg/mmol creatinine) over a range of possible clinical conditions. The best statistical cut-off value of Fetuin-A (μg/mmol creatinine) was defined as the point at which the sum of sensitivity and specificity is more than that at other points. GraphPad Prism v6 was used.

## Additional files

Additional file 1: Figure S1. Absence of Fetuin-A staining in the medulla and in renal pelvis. Additional file 2: Table S1. Clinical characteristics of patients with other renal diseases. Additional file 3: Figure S2. Fetuin-A expression levels in urine samples. Additional file 4: Figure S3. Evolution of Fetuin-A and eGFR in ADPKD patients.

## Abbreviations

AC6: Adenylate cyclase-6
ADPKD: Autosomal dominant polycystic kidney disease
AKI: Acute kidney injury
AUC: Area under the curve
BP: Blood pressure
CKD: Chronic kidney disease
CT-scan: Computed tomography scan
eGFR: Estimated glomerular filtration rate
ELISA: Enzyme-linked immunosorbent assay
ESRD: End stage renal disease
kDa: Kilodalton
KO: Knock-out
LTL: *Lotus tetragonolobus* lectin
MRI: Magnetic resonance imaging
N.A.: Not assessed
n.s.: Not significant
OCT: Optimum cutting temperature
PBS: Phosphate-buffered saline
PBS-T: Phosphate-buffered saline tween-20
ROC: Receiving operating curve
SDS-PAGE: Sodium dodecyl sulfate polyacrylamide gel electrophoresis
U: Urine
WT: Wild-type
