# Supplementary material for: Urine Fetuin-A is a biomarker of autosomal dominant polycystic kidney disease progression
Source: J Transl Med. 2015 Mar 30;13:103. doi: 10.1186/s12967-015-0463-7 (PMC4416261; doi:10.1186/s12967-015-0463-7)
Supplement: Additional file 1: Figure S1. — Absence of Fetuin-A staining in the medulla and in renal pelvis. [file 12967_2015_463_MOESM1_ESM.pdf]

Additonal file 1: Figure S1

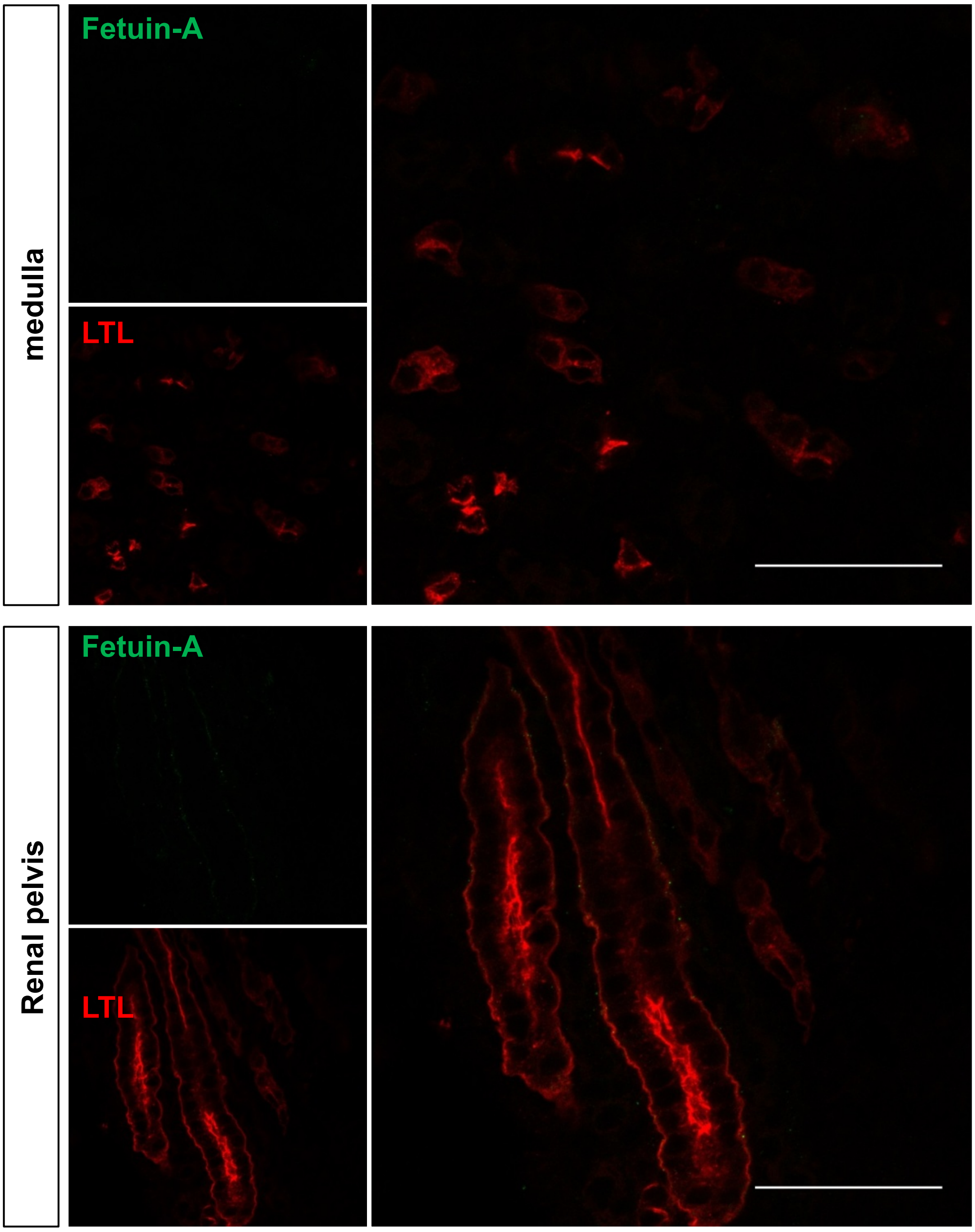

Additional file 1: Figure S1. Absence of Fetuin-A staining in the medulla and in renal pelvis
Frozen sections of WT newborn kidneys labeled with anti-Fetuin-A antibody (green), and *Lotus tetragonolobus* lectin (LTL, red). Scale bars: 100 µm.
