# Supplementary material for: Urine Fetuin-A is a biomarker of autosomal dominant polycystic kidney disease progression
Source: J Transl Med. 2015 Mar 30;13:103. doi: 10.1186/s12967-015-0463-7 (PMC4416261; doi:10.1186/s12967-015-0463-7)
Supplement: Additional file 2: Table S1. — Clinical characteristics of patients with other renal diseases. [file 12967_2015_463_MOESM2_ESM.docx]

**Additonal file S2: Table S1. Clinical characteristics of patients with other renal diseases**

| Causes of renal insufficiency | n (%) |
| --- | --- |
| Vascular (hypertension, nephroangiosclerosis, …)  Glomerular (glomerulonephritis, FSGS, diabetes, lupus,…)  Tubulointerstitial  Others | 26 (38%)  19 (28%)  17 (24%)  7 (10%) |
