# Supplementary material for: Urine Fetuin-A is a biomarker of autosomal dominant polycystic kidney disease progression
Source: J Transl Med. 2015 Mar 30;13:103. doi: 10.1186/s12967-015-0463-7 (PMC4416261; doi:10.1186/s12967-015-0463-7)
Supplement: Additional file 3: Figure S2. — Fetuin-A expression levels in urine samples. [file 12967_2015_463_MOESM3_ESM.pdf]

Additonal file 3: Figure S2

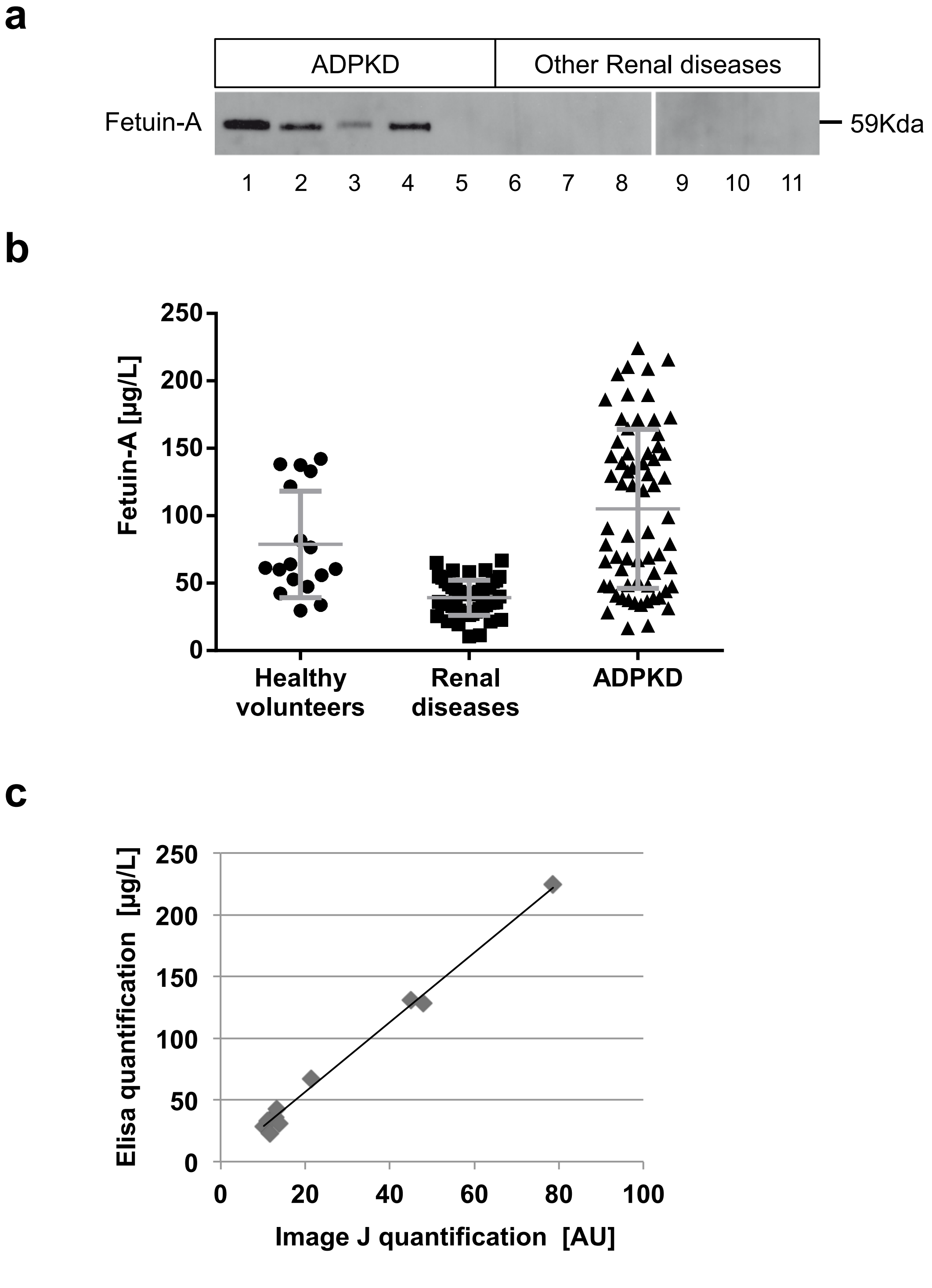

Additional file 3. Figure S2. Fetuin-A expression levels in urine samples
(a) Western blot analysis of Fetuin-A in urine samples of ADPKD patients (n = 5) and of patients with renal diseases other than ADPKD (n = 6). **(b)** ELISA quantification of Fetuin-A levels in urine of ADPKD (n = 66), healthy volunteers (n = 17) and control patients with various renal diseases (n = 50). Solid lines indicate mean. **(c)** Correlation between western blot and ELISA analyses. Line shows linear regression (r = 0.992).
