# Supplementary material for: Urine Fetuin-A is a biomarker of autosomal dominant polycystic kidney disease progression
Source: J Transl Med. 2015 Mar 30;13:103. doi: 10.1186/s12967-015-0463-7 (PMC4416261; doi:10.1186/s12967-015-0463-7)
Supplement: Additional file 4: Figure S3. — Evolution of Fetuin-A and eGFR in ADPKD patients. [file 12967_2015_463_MOESM4_ESM.pdf]

Additonal file 4: Figure S3

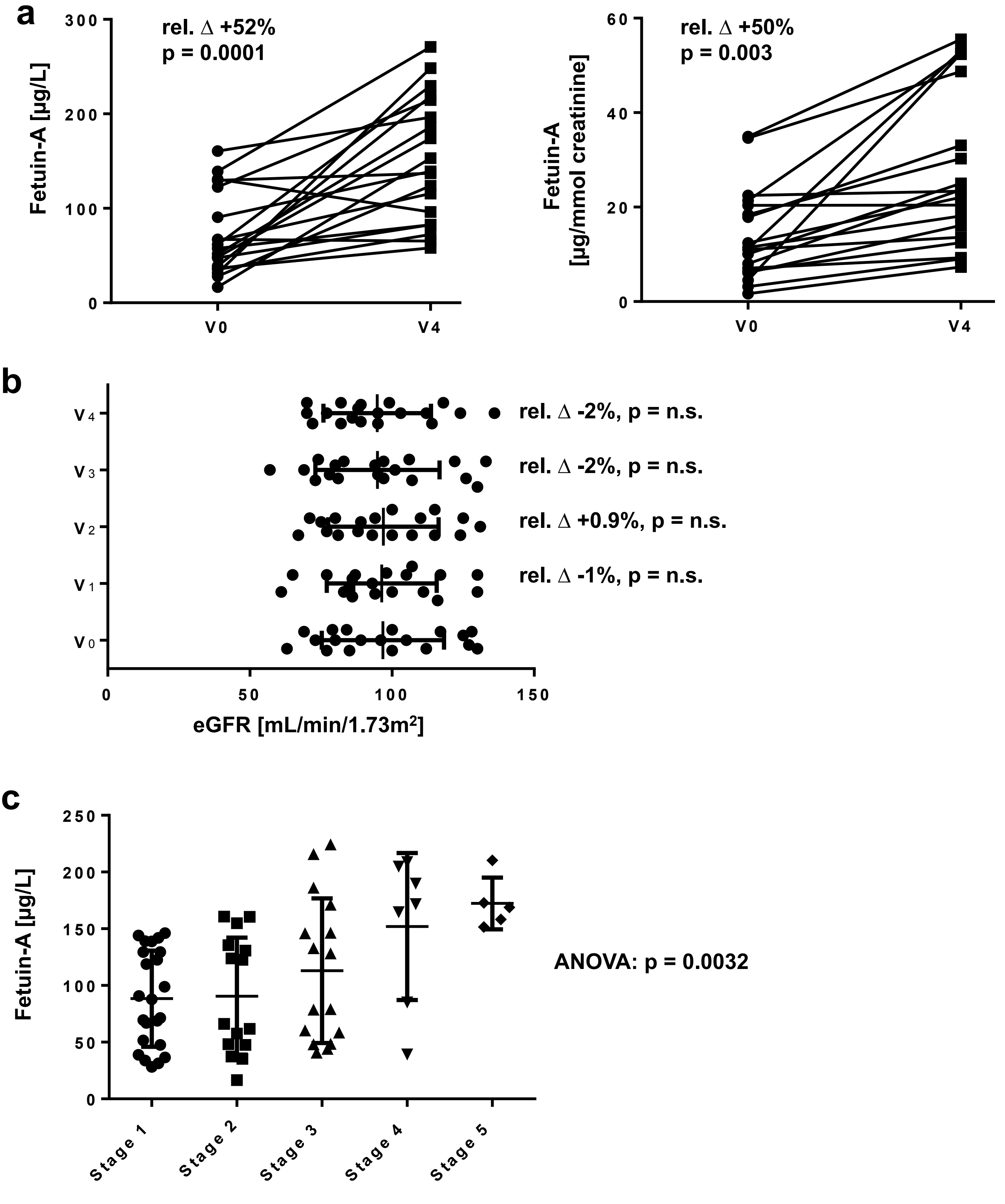

Additonal file 4: Figure S3. Evolution of Fetuin-A and eGFR in ADPKD patients
(a, b) Evolution of urinary levels of Fetuin-A and eGFR during 5 visits (V) every 6 months in 19 early ADPKD patients. V_0: first visit; V_4: last visit. Average relative change to initial values (Δ, %) and p-values are indicated for each analysis. **(c)** Urinary levels of Fetuin-A stratified by CKD stages. Stage 1, n = 23; stage 2, n = 15; stage 3, n = 16; stage 4, n = 7 and stage 5, n = 5. Solid lines indicate mean. p-value = 0.0032 (one-way ANOVA).
